# Epidemiologic features of child unintentional injury in rural PuCheng, China

**DOI:** 10.5249/jivr.v5i2.304

**Published:** 2013-06

**Authors:** Shaohua Li, Zhiru Tang, Xiujun Zhang, Lilun Yan, Shidong Wang, Guoqi Liu, Guo Zhang, Mingxing Zhu, David C. Schwebel, Yehuan Sun

**Affiliations:** ^*a*^Anhui Medical University, Hefei City, PR China.; ^*b*^Department of Psychology, University of Alabama at Birmingham, USA.

**Keywords:** Injury, Safety, Rural, Children, China

## Abstract

**Background:**

Epidemiologic features of unintentional injuries among children in rural China are unknown.

**Methods:**

Using a stratified sampling method, we conducted a retrospective study of injury reports over a year-long period. Structured oral questionnaires were administered to parents of 2551 children ages 0 to 14.

**Results:**

The annual incidence of unintentional injuries was 15.6%, with boys (16.7%) having a slightly higher rate than girls (14.5%; p greater than .05). The four leading causes of injury for both genders were falls (5.1% annual incidence), burns (2.9%), animal-related injuries (1.7%), and traffic injuries (1.6%).

**Conclusions:**

Unintentional injuries have substantial negative effects on children and their families. In rural PuCheng, China, the incidence of unintentional injury among children is extremely high compared to other regions of the world. The types of injuries incurred are somewhat different from those reported in other geographic areas. Injury prevention programs targeting the issues specific to this region, and similar rural regions of China, are needed.

## Introduction

Worldwide, unintentional injuries account for about 5 million deaths annually, with more than 90 percent of unintentional injury deaths occurring in low- and middle-income countries.^1^ In China, unintentional injury incidence rate estimates range from 11.34% to 13.86%,^2,3^ depending on geographic region, with estimates in rural areas slightly higher than those in urban cities.^4^ Unintentional injuries account for about 60% of all deaths to Chinese children ages 0 to 14.^5^ Of course, morbidity rates far exceed mortality and also have enormous impact on children and their families.

Children in rural areas of China have particular risk of unintentional injury for a number of reasons, including exposure to work-related hazards, exposure to open water, animals, and other natural hazards, and inadequate supervision by parents.^6^ Poor children are more likely to live in rural areas, and also have poor access to health care and insufficient funds to pay for care.

Previous work on the epidemiology of childhood injury in rural China is limited. In one study of several districts in JiangSu province in Southern China, drowning was reported as the primary cause of death in children ages 0 to 14, with a mortality rate of 73.0/100,000, 36 times the rate of drowning in urban areas.^7^ Others have examined injury rates at schools^8^ and fatal deaths across the lifespan,^4^ but very little work has examined non-fatal pediatric injury epidemiology in rural China.

This study was designed to provide further information about nonfatal unintentional child injury rates in rural China. Knowledge about such injury rates is essential to develop appropriate injury prevention strategies targeting at-risk children and situations.

## Methods

**District**

The research was conducted in PuCheng district, Shanxi province, China. Shanxi province is located in North Central China and is a predominantly agricultural region. Wheat is the primary crop. PuCheng district covers an area of 1,584 square miles within Shanxi province and had a population of approximately 745,000 people in the 2005 census. This population included about 180,000 children under age 18 (24% of population), about 136,000 of whom attended 41 high schools, 315 primary schools, and 45 kindergartens. At the time of the study in 2008, the approximate per capita net income of farmers in the region was $280 US dollars and of families in semi-urban areas of the district it was $1030 dollars. About 87% of the district’s population was farmers.

**Participants**

Data were collected in May 2008 using a stratified sampling method. We first randomly selected three towns in PuCheng for inclusion. Next, we selected from those towns, at random, one middle school, two primary schools, two preschools, and individual families with infants and toddlers. This strategy yielded a total sample of 2640 children, 2551 of whose parents completed usable surveys (96.6%). Of the 2551 children, 1297 boys (50.8%) and 1254 girls (49.2%) participated. The sample included 550 infants and toddlers ages 0-2 (21.6%), 534 preschoolers ages 3-5 (20.9%), 942 elementary school children ages 6-11 (36.9%), and 525 middle school children ages 12-14 (20.6%). The sample approximates but does not match precisely the distribution of population of children under age 14 in PuCheng District.

**Protocol**

Data collection was conducted using a standard protocol by carefully-trained research staff. Standard informed consent procedures were completed first with interviewees. Once consent was given, a short face-to-face structured interview was conducted with the parent. Children were present during the interview to assist parents in answering questions if necessary.

After the data collection, a database of injury reports was constructed using EpiData3.1. Partial double entry was implemented by trained staff to ensure accuracy. Statistical analyses were conducted in SPSS 10.0.

**Measures**

The primary measure of interest was frequency of unintentional injury over the prior year (April 2007-April 2008). Previous research indicates the reliability and validity of parent recall of such major injuries.^9-10^ Injuries were defined to parents as hospital-diagnosed accidents resulting in emergency treatment and/or discontinuation of the child’s normal activities for at least half a day. This classification represents a fairly major injury and is consistent with definitions in the International Classification of Disease.

For each injury reported, a structured protocol was used to evaluate information on the environment where the injury occurred, the type of injury, and the treatment used.

## Results

**General information about injury incidence**

We surveyed 2551 children. Thirteen percent of the sample (N = 332) reported one or more injuries, with 281 children reporting one injury, 36 two injuries, and 15 three injuries.

Table 1 lists the injury incidence rate of the overall sample, as well as the gender and age-based groups studied. As shown, the overall incidence of unintentional injuries was 15.6%, with boys (16.7%) having a slightly higher rate than girls (14.5%). For both boys and girls, the incidence of unintentional injuries for children aged 1 to 2 (25.7%) and ages 12-14 (24.0%) were the highest, with incidence among children aged 0 to 1 (3.0%) the lowest.

**Table 1:** Incidence of unintentional injury among gender and age groups

Age	Male			Female			Total			p (X^2^)
	n	Injuries	Incidence(%)	n	Injuries	Incidence(%)	n	Injuries	Incidence(%)	
<1	81	2	2.5	52	2	3.8	133	4	3.0	0.66
1-2	214	50	23.4	203	57	28.1	417	107	25.7	0.40
3-5	284	46	16.2	250	39	15.6	534	85	15.9	0.87
6-8	236	29	12.3	200	11	5.5	436	40	9.2	0.03
9-11	247	24	9.7	259	12	4.6	506	36	7.1	0.03
12-14	235	65	27.7	290	61	21.0	525	126	24.0	0.17
Total	1297	216	16.7	1254	182	14.5	2551	398	15.6	0.20

**Injury characteristics**

Table 2 illustrates the incidence of various types of injuries. The incidence of falling was highest (5.1%, and 32.9% of all injuries reported), with boys (5.4%) having a higher rate of falls than girls (4.9%). Followed by falls, the most common causes of injuries were burns (18.6% of injuries reported), animal-related bites (10.8%), road traffic injuries (10.1%), injuries from sharp instruments (6.8%), and hitting injuries (6.0%). Burns encompassed all injuries caused by heat or fire, including burns from steam, food, fire, and so on. Animal-related bites included bites from both domesticated animals, especially from dogs, as well as from non-domesticated animals including stray dogs and cats, rats, and so on. Road traffic injuries included a range of pedestrian, bicycling, and motor vehicle injuries; most common were bicycling injuries incurred while children traveled to and from school. Other injuries (14.8%) encompassed a wide range of injury types, including being struck by/against, extrusions, poisonings, burns (primarily by fireworks), and environmental injuries (including weather-related injury such as sunstroke and frostbite as well as machinery-related injury such as those incurred from sickles and tractors).

**Table 2:** The incidence (%) of types of injury among gender groups

Type of Injury	Male		Female		Total		Composition (%)
	Injuries	Incidence	Injuries	Incidence	Injuries	Incidence	
Falls	70	5.4	61	4.9	131	5.1	32.9
Burns	37	2.9	37	3.0	74	2.9	18.6
Animal Related	27	2.1	16	1.3	43	1.7	10.8
Traffic	24	1.9	16	1.3	40	1.6	10.1
Sharp Instrument	13	1.0	14	1.1	27	1.1	6.8
Hitting	13	1.0	11	0.9	24	0.9	6.0
Other	32	2.5	27	2.2	59	2.3	14.8
Total	216	16.8	182	14.7	398	15.6	100.0

**Distribution of the locations of injury occurrence**

As one might expect, children's unintentional injuries occurred in various places, including at home (51.8%), at school but outside the classroom (14.8%), outside the home residence (14.1%), while traveling to and from school (7.3%), and in other places (8.0%) including the classroom (4.0%) (See Table 3). The most frequent locations for injuries, not surprisingly, were the places where children spend most of their time and where the greatest risks are present. 

**Table 3:** Distribution of injury occurrence locations

Location of Injury	Male		Female		Total	Composition (%)
	Times	Incidence (%)	Times	Incidence (%)		
At home	113	8.7	93	7.4	206	51.8
Outside classroom,Within school	31	2.4	28	2.2	59	14.8
Outside, but around home	26	2.0	30	2.4	56	14.1
Between home and school	20	1.5	9	0.7	29	7.3
At classroom	9	0.7	7	0.6	16	4.0
Other	17	1.3	15	1.2	32	8.0

## Discussion

These data from PuCheng District, a predominantly agricultural wheat-producing region of Northern China, are valuable for scientists to understand the scope of the nonfatal unintentional child injury problem in rural China and to begin to consider ways to intervene to prevent such injuries. Our findings suggest the incidence of unintentional injuries among children in this rural area of China was 15.6%, with boys (16.7%) having a slightly higher rate than girls (14.5%). The gender difference was not statistically significant. The four leading causes of injury for both genders were falls (5.1%), burns (2.9%), animal-related injuries (1.7%), and traffic injuries (1.6%). 

The 15.6% prevalence rate for non-fatal pediatric injury is higher than that reported in other regions of the world. In the United States, for example, the WISQARS database reports a prevalence of 10.2% for nonfatal injuries requiring professional medical attention (a lower threshold for injury identification than used in the present study). Pakistan, a developing nation economically comparable to China, has an incidence rate of non-fatal injuries requiring care outside the home for children aged 1-8 years of 12.1% in rural areas.^11^ It is unclear why the rate may be higher in rural China than in other regions, although environmental risks (poorly maintained roads and sidewalks, exposure to animals), cultural practices (parents busy with agricultural tasks and not supervising children), and access to medical care (the Chinese health care system may be more accessible/advanced than the Pakistani one) are leading explanatory hypotheses to explore in future research.

The finding that falls was the leading cause of injury was intriguing. Traffic-related injuries are generally reported as the leading cause of injury in most rural (and urban) areas of the world, including in low- and middle-income nations,^12-14^ but there is other evidence that falls are a leading cause of non-fatal injury in China.^15-18^ It may be that risk in China differs from other nations; one contributor that might be considered in future research is the possibility that rural areas of China have comparatively fewer vehicles and travel on the roads, whereas children in those areas are prone to fall or injure themselves in other activities. Also relevant is the high use of walking and bicycling in rural China, different from some other nations where automobile transport is more common.

Another intriguing aspect of our results was the gender differences in injury incidence. The incidence of most injury types, including falls, animal-related injuries, and traffic-related injuries, were more common among boys, although usually by a small and not statistically significant margin. The overall incidence of injuries among boys was 16.7% and among girls 14.5%, creating a ratio of 1.15:1 between boys and girls. In most other nations, including the USA, the ratio of the incidence of unintentional injuries between boys and girls is higher, ranging from 2.05:1 to 3.34:1.^19^ It may be that the girls in rural China are exposed to more risk, as they are frequently active and engaged in housework and fieldwork; future research should investigate whether this cultural practice might influence injury risk. Interestingly, the rate of burns was higher in girls (ratio of 0.97:1 for boys: girls). This pattern has been seen in other cultures,^20^ and may be a result of the fact that girls spend more time in the home than boys, where they are exposed to risk from kerosene heaters and to hot liquids and foods while cooking.

Our study was conducted in a rural area of a middle-income nation, China. Various authorities and scientists have emphasized the need for injury prevention among such vulnerable individuals and regions^1,20^ because those regions are understudied and underserved in injury prevention, yet overrepresented in injury rates. These data confirm that fact, demonstrating the high rate of injuries compared to other regions studied in the past.^11^

Our study examined non-fatal injuries. The larger portion of epidemiological injury research examines fatal injuries. Understanding non-fatal injury rates is important given the emotional and financial toll non-fatal injuries can place on families. Non-fatal childhood injuries impose a significant burden on morbidity worldwide; 400 million children are estimated to suffer from non-fatal injuries every year.^21^ Non-fatal injuries may lead to serious permanent disability and disfigurement and can impact and impede children’s development.^22^ Plus, the scope of the problem in China is enormous given the country’s large population. National estimates indicate about 40 million Chinese children suffer unintentional injuries annually, including 13.6 million who receive outpatient hospital care, 3.4 million who need emergency treatment in the hospital, 1.27 million students who lose normal functioning capacity temporarily, and 340,000 who suffer permanent disabilities (as a means of comparison, 74 million children total live in the USA, the most populous high-income nation in the world).

**Implications**

A large body of evidence documents the multiple intrapsychic, interpersonal, and environmental causes of children’s unintentional injuries.^23^ By identifying the types of injuries that occur in rural locations of low and middle income countries such as PuCheng District, China, we can begin to consider ways to identify and change modifiable risk factors. Among the areas that might be considered for intervention are physical and mental development of the children, educating or training the family, and making children and parents more aware of potential risks in their environment.

**Limitations**

Before concluding, we mention limitations of this research. The study covered only one rural district in China and spanned only one year of observation. Because of this, our results may not generalize to other districts or time periods. Further, we recruited a large sample of children but the sample is not necessarily proportional to children’s ages in the population of PuCheng district. Therefore, assumptions about population-based injury rates must be made cautiously. A second limitation is that we relied on self-report data. Although we expect results were validly and reliably reported by the participants, we do not have evidence of this fact. Third, we have only basic information on the injuries that were reported by our sample. Future research would benefit from details about the context and severity of injuries, including preventable behavioral and environmental factors that may have led to the injury incident and could be altered to prevent future or similar events. Finally, our study focused only on children ages 0-14. Future work might consider injury risk to adolescents and to various groups of adults.

## Conclusion

In conclusion, we found child unintentional injury rates vary among different age groups and genders in rural PuCheng County, China. We hope our findings lead to development of effective interventions that can be extended broadly to rural populations in China and other developing nations.
